# Avutometinib

**DOI:** 10.1107/S2056989026004482

**Published:** 2026-05-15

**Authors:** Jacob K. Salazar, James A. Kaduk, Anja Dosen, Thomas N. Blanton

**Affiliations:** a North Central College, Department of Chemistry 131 S Loomis St Naperville IL 60540 USA; b North Central College, Department of Physics 131 S Loomis St Naperville IL 60540 USA; c Illinois Institute of Technology, Department of Chemistry, 3101 S. Dearborn St., Chicago IL 60616, USA; d ICDD, 12 Campus Blvd., Newtown Square PA 19073-3273, USA; Universidade Federal do ABC, Brazil

**Keywords:** powder diffraction, avutometinib, AVMAKI, Rietveld refinement, density functional theory, crystal structure

## Abstract

The crystal structure of avutometinib (systematic name: 3-{[3-fluoro-2-(methyl­sulfamoyl­amino)­pyridin-4-yl]meth­yl}-4-methyl-7-(pyrimidin-2-yloxy)­chro­men-2-one), C_21_H_18_FN_5_O_5_S, has been solved and refined using synchrotron X-ray powder diffraction data, and optimized using density functional theory techniques. Avutometinib crystallizes in space group *P*1 (#2). The crystal structure is composed of layers parallel to the *ab* plane. N—H⋯O hydrogen bonds link the layers along the *a*-axis direction. The mol­ecule is Z-shaped.

## Abstract 2

The crystal structure of avutometinib has been solved and refined using synchrotron X-ray powder diffraction data, and optimized using density functional theory techniques.

## 1. Chemical context

Avutometinib (AVNAPKI) has been approved as a treatment for ovarian cancer. AVNAPKI is administered in capsule form as a co-medication with FAKZYNJA™ (defactinib tablets), for the treatment of KRAS-mutated recurrent low-grade serous ovarian cancer for patients that have previously received unsuccessful systemic therapy. The systematic name (CAS Registry Number 946128-88-7) is 3-{[3-fluoro-2-(methyl­sulfamoyl­amino)-4-pyridin­yl]meth­yl}-4-methyl-7-pyrimidin-2-yloxychromen-2-one.

We are unaware of any published powder diffraction data for avutometinib. This work was carried out as part of a project (Kaduk *et al.*, 2014 ▸) to determine the crystal structures of large-volume commercial pharmaceuticals, and include high-quality powder diffraction data for them in the Powder Diffraction File (Kabekkodu *et al.*, 2024 ▸).
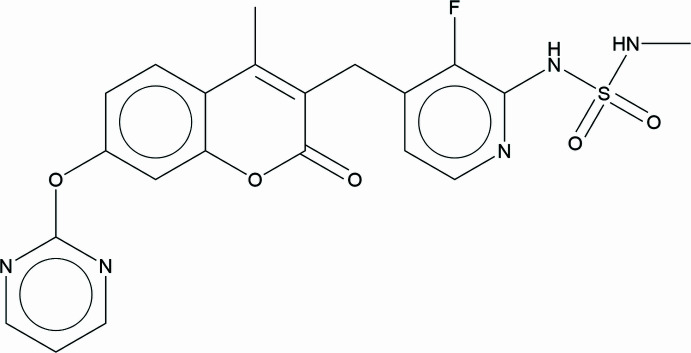


## 2. Structural commentary

**Figure 1:**
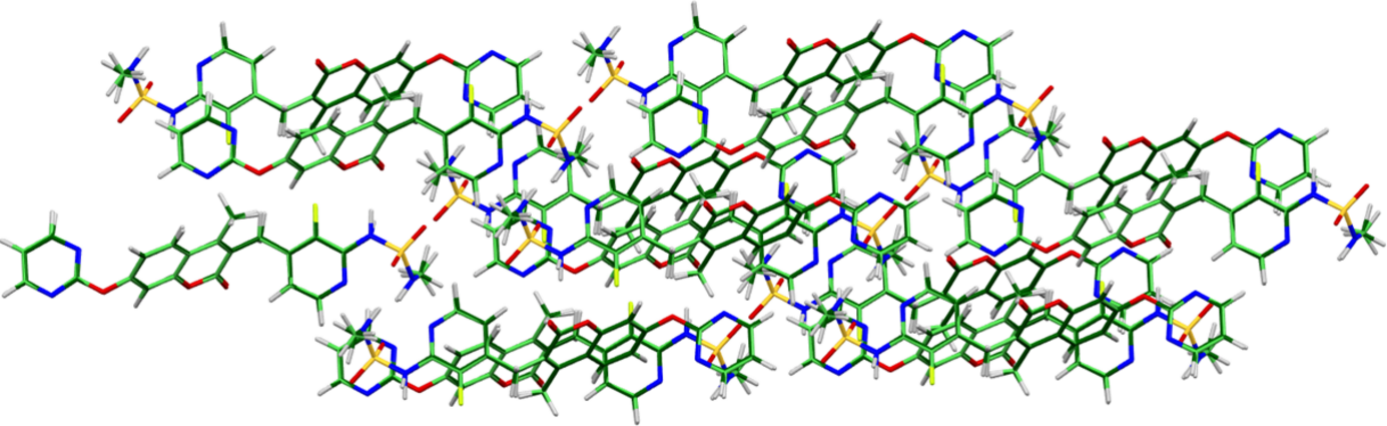
Comparison of the Rietveld-refined (colored by atom type) and VASP-optimized (pale green) structures of avutometinib, calculated using the *Mercury* CSD-Materials/Search/Crystal Packing Similarity tool. The root-mean-square Cartesian displacement is 0.059 Å. Image generated using *Mercury* (Macrae *et al.*, 2020 ▸).

**Figure 2:**
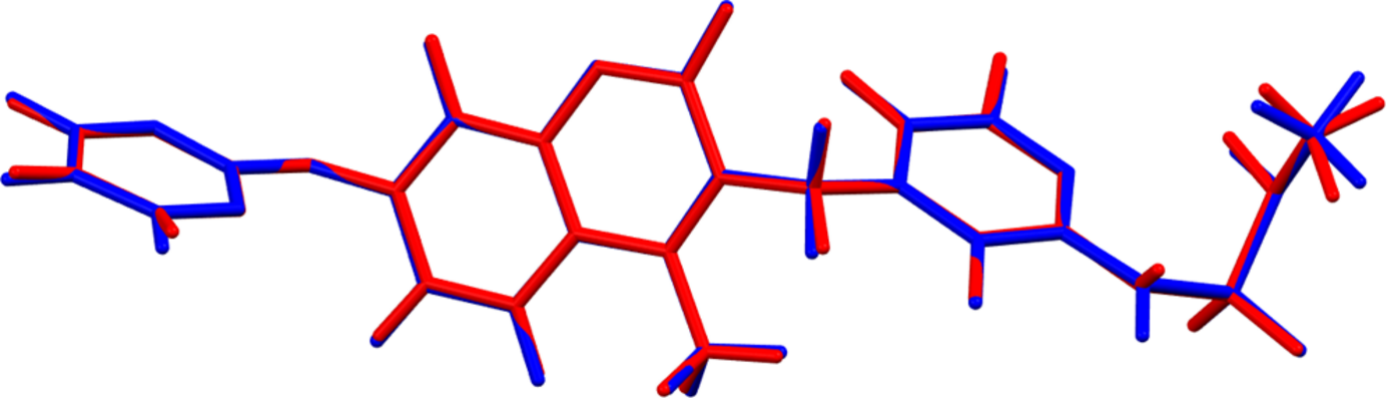
Comparison of the refined structure of avutometinib (red) to the VASP-optimized structure (blue). The comparison was generated using the *Mercury* Calculate/Mol­ecule Overlay tool; the r.m.s. difference is 0.053 Å. Image generated using *Mercury* (Macrae *et al.*, 2020 ▸).

**Figure 3:**
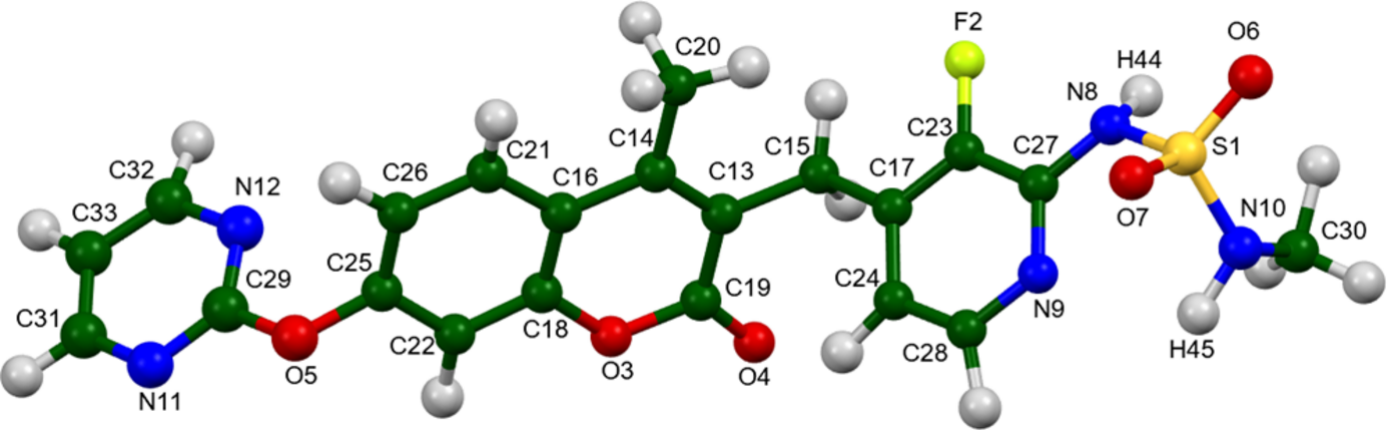
The asymmetric unit of avutometinib, with the atom numbering. The atoms are represented by 50% probability spheroids. Image generated using *Mercury* (Macrae *et al.*, 2020 ▸).

The root-mean-square difference of the non-H atoms in the Rietveld-refined and VASP-optimized structures of avutometinib, calculated using the *Mercury* (Macrae *et al.*, 2020 ▸) CSD-Materials/Search/Crystal Packing Similarity tool is 0.059 Å (Fig. 1 ▸); the structures are essentially identical. The root-mean-square Cartesian displacement of the non-H atoms in the refined and optimized structures, calculated using the *Mercury* Calculate/Mol­ecule Overlay tool, is 0.053 Å (Fig. 2 ▸). The agreements are within the normal range for correct structures (van de Streek & Neumann, 2014 ▸). The asymmetric unit is illustrated in Fig. 3 ▸. The remaining discussion will emphasize the VASP-optimized structure.

All of the bond distances, bond angles, and torsion angles fall within the normal ranges indicated by a *Mercury* Mogul Geometry check (Macrae *et al.*, 2020 ▸). Only the S1—N8 bond of 1.682 Å [average = 1.628 (17); *Z*-score = 3.1] is flagged as unusual. The unusual S—N bond distance is an example of a known feature of DFT calculations: too-long S—N bonds in the DFT optimization of sulfonamides have been observed (Kaduk *et al.*, 2025 ▸; Vibha *et al.*, 2023 ▸; Whitfield, 2025 ▸).

Quantum chemical geometry optimization of the isolated avutometinib mol­ecule (DFT/B3LYP/6-31G*/water) using *Spartan ’24* (Wavefunction, 2025 ▸) indicated that the observed conformation is 5.7 kcal mol^−1^ higher in energy than a local minimum, which has a very similar conformation. The global minimum-energy conformation is 79.9 kcal mol^−1^ lower in energy, but is folded on itself to make intra­molecular hydrogen bonds. Inter­molecular inter­actions are thus important in determining the observed solid-state conformation.

## 3. Supra­molecular features

**Figure 4:**
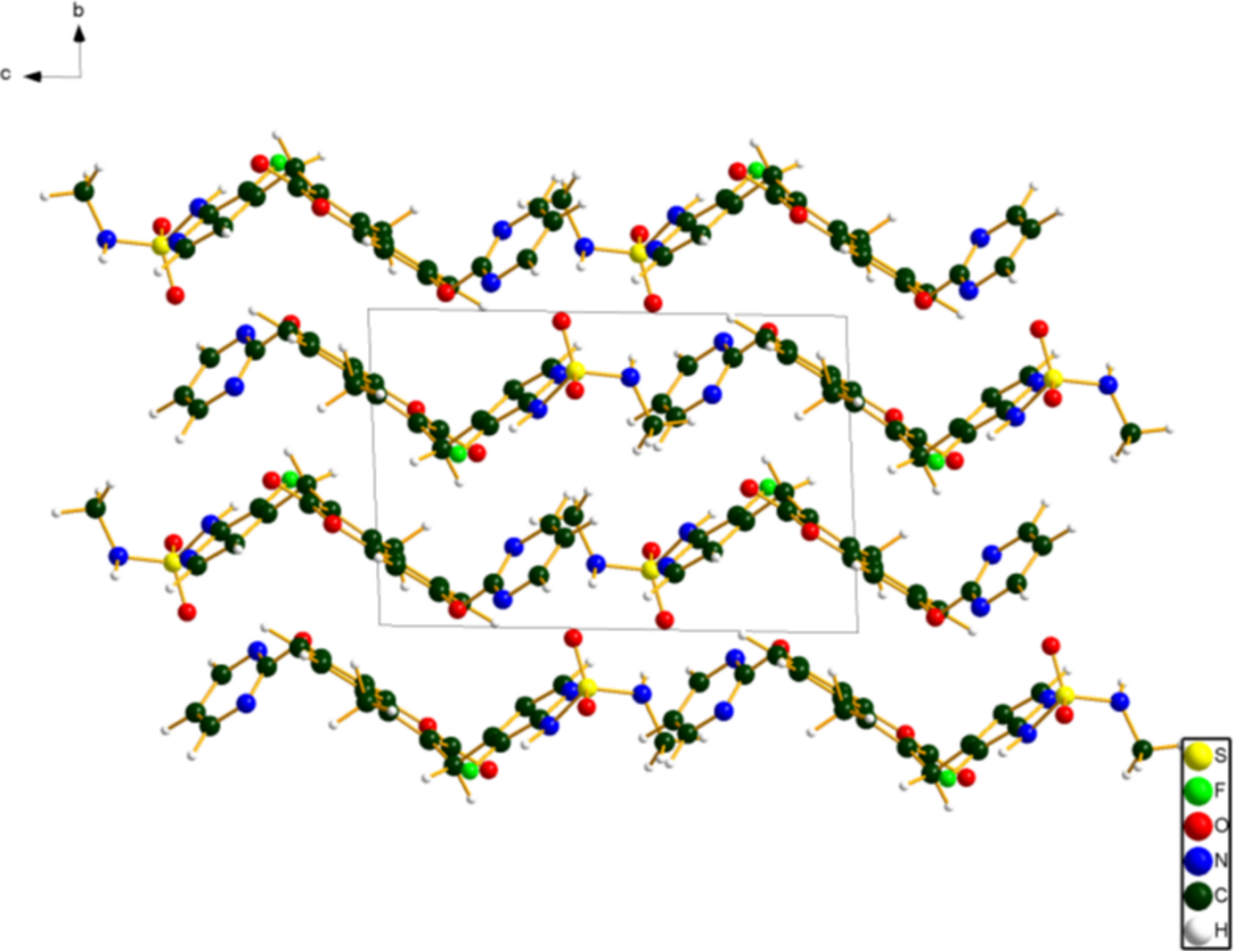
Crystal structure of avutometinib, viewed down the *a*-axis. Image generated using DIAMOND (Brandenburg & Putz, 2025 ▸).

The crystal structure (Fig. 4 ▸) is composed of layers lying parallel to the *ab* plane. Hydrogen bonds link the layers along the *b*-axis direction (Table 1 ▸). The mol­ecule is Z-shaped. The mean plane of the pyridine ring near the sulfonamide group is approximately (011), the mean plane of the 2*H*-chromen-2-one ring system is approximately (01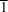), and the mean plane of the pyrimidine ring is approximately (123). The *Mercury* Aromatics Analyser indicates one strong inter­action (*d* = 4.334 Å) between phenyl rings of the 2*H*-chromen-2-one ring system, and weaker inter­actions between multiple pairs of rings.

Analysis of the contributions to the total crystal energy of the structure using the Forcite module of *Materials Studio* (Dassault Systèmes, 2024 ▸) indicated that the intra­molecular energy is dominated by angle distortion terms, as might be expected for a mol­ecule containing a fused ring system. The inter­molecular energy is dominated by van der Waals attractions, which in this force field based analysis include hydrogen bonds. The hydrogen bonds are better discussed using the results of the DFT calculation.

There are two classical N—H⋯O hydrogen bonds in the structure (Table 1 ▸), one intra­molecular and one inter­molecular. The energies of these hydrogen bonds were calculated using the correlation of Wheatley and Kaduk (2019 ▸). The inter­molecular N8—H44⋯O4 hydrogen bonds link the mol­ecules into chains along the *a*-axis direction, with graph set *C*^1^_1_(9) (Etter, 1990 ▸; Bernstein *et al.*, 1995 ▸; Motherwell *et al.*, 2000 ▸). A few C—H⋯O and C—H⋯N hydrogen bonds also contribute to the cohesion of the crystal.

**Figure 5:**
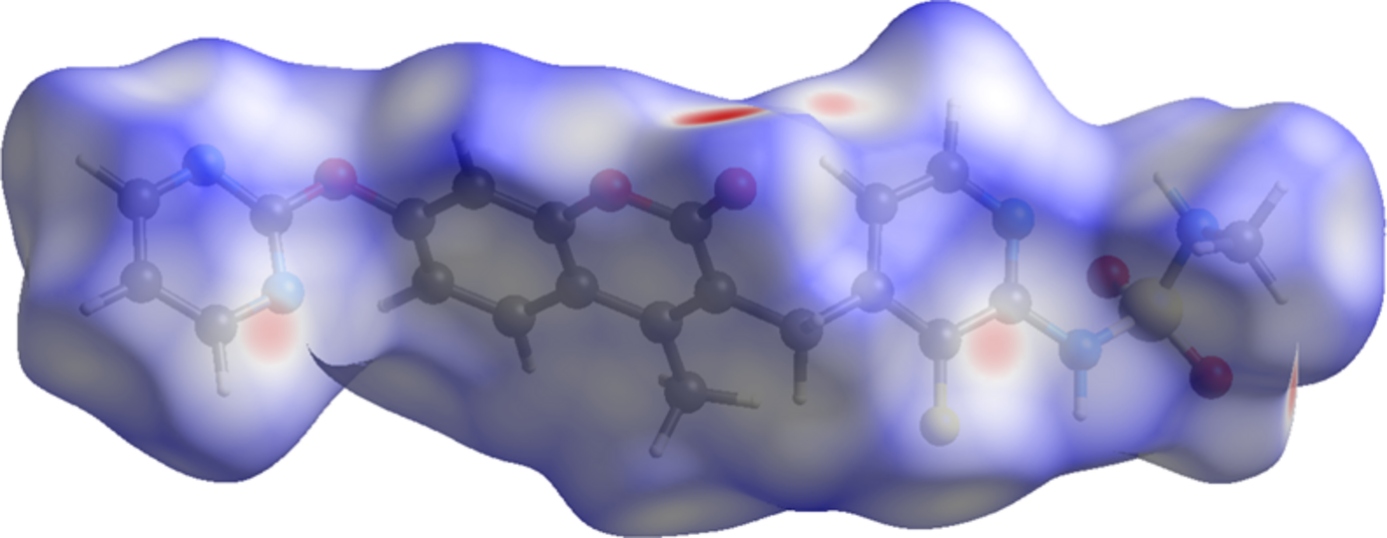
The Hirshfeld surface of avutometinib. Inter­molecular contacts longer than the sums of the van der Waals radii are colored blue, and contacts shorter than the sums of the radii are colored red. Contacts equal to the sums of radii are white. Image generated using *CrystalExplorer* (Spackman *et al.*, 2021 ▸).

The volume enclosed by the Hirshfeld surface of avutometinib (Fig. 5 ▸, Hirshfeld, 1977 ▸; Spackman *et al.*, 2021 ▸) is 498.97 Å^3^, 98.27% of half of the unit-cell volume. The packing density is thus typical. The only significant close contacts (red in Fig. 5 ▸) involve the hydrogen bonds. The volume/non-hydrogen atom is smaller than normal, at 15.4 Å^3^.

The Bravais–Friedel–Donnay–Harker (Bravais, 1866 ▸; Friedel, 1907 ▸; Donnay and Harker, 1937 ▸) algorithm suggests that we might expect lozenge morphology for avutometinib, with {001} as the major faces. A 2^nd^-order spherical harmonic model for preferred orientation was included. The texture index was 1.002, indicating that the preferred orientation was insignificant in this rotated capillary specimen.

## 4. Database survey

A reduced cell search of the Cambridge Structural Database (Groom *et al.*, 2016 ▸) yielded no hits.

## 5. Synthesis and crystallization

Avutometinib was a commercial reagent, purchased from Sigma (Batch #A245684-HA3), and was used as-received.

## 6. Refinement

Crystal data, data collection and structure refinement details are summarized in Table 2 ▸. The white powder was packed into a 1.5 mm diameter Kapton capillary, and rotated during the measurement at ∼50 Hz. The powder pattern was measured at 295 K at beam line 11-BM (Lee *et al.*, 2008 ▸; Wang *et al.*, 2008 ▸; Antao *et al.*, 2008 ▸) of the Advanced Photon Source at Argonne National Laboratory using a wavelength of 0.4687342 Å from 0.5–50° 2θ with a step size of 0.001° and a counting time of 0.1 sec/step. The high-resolution powder diffraction data were collected using twelve silicon crystal analyzers that allow for high angular resolution, high precision, and accurate peak positions. A mixture of silicon (NIST SRM 640c) and alumina (NIST SRM 676a) standards (ratio Al_2_O_3_:Si = 2:1 by weight) was used to calibrate the instrument and refine the monochromatic wavelength used in the experiment.

The pattern was indexed on a high-quality primitive triclinic unit cell with *a* = 8.91271, *b* = 9.47911, *c* = 13.29736 Å, *α* = 83.91, *β* = 81.66, *γ* = 66.67°, *V* = 1019.23 Å^3^, and *Z* = 2 using *JADE Pro* (MDI, 2025 ▸). The space group was assumed to be *P*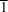, which was confirmed by successful solution and refinement of the structure.

The mol­ecular structure of avutometinib was downloaded from PubChem (Kim *et al.*, 2023 ▸) as Conformer3D_COMPOUND_CID_16719221.sdf. It was converted to a *.mol2 file using *Mercury* (Macrae *et al.*, 2020 ▸), and to a Fenske–Hall *Z*-matrix using *OpenBabel* (O’Boyle *et al.*, 2011 ▸). The structure was solved using parallel tempering techniques as implemented in *FOX* (Favre-Nicolin & Černý, 2002 ▸).

Rietveld refinement was carried out using *GSAS-II* (Toby & Von Dreele, 2013 ▸). Only the 1.9–30.0° portion of the pattern was included in the refinements (*d*_min_ = 0.905 Å). The μ*R* value was fixed at 0.02, calculated using the 11-BM web site (https://11bm.xray.aps.anl.gov/absorb/). All non-H bond distances and angles were subjected to restraints, based on a *Mercury*/Mogul Geometry Check (Sykes *et al.*, 2011 ▸; Bruno *et al.*, 2004 ▸). The Mogul average and standard deviation for each qu­antity were used as the restraint parameters. The aromatic rings were restrained to be planar. The restraints contributed 3.7% to the overall *χ^2^*. The hydrogen atoms were included in calculated positions, which were recalculated during the refinement using *Materials Studio* (Dassault Systèmes, 2024 ▸). The *U*_iso_ values were grouped by chemical similarity. The peak profiles were described using the generalized microstrain model (Stephens, 1999 ▸). The background was modeled using a six-term shifted Chebyshev polynomial, with a peak at 5.87° 2θ to model the scattering from the Kapton capillary and any amorphous component of the sample.

**Figure 6:**
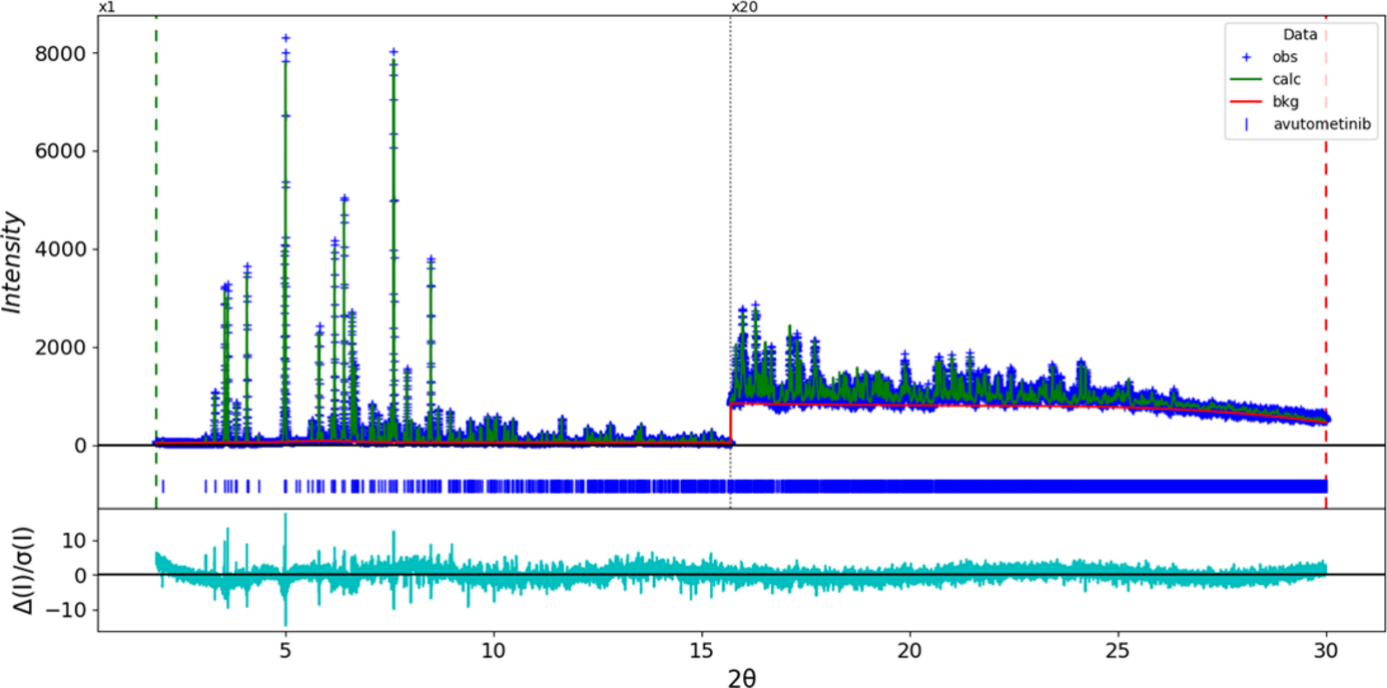
The Rietveld plot for avutometinib. The blue crosses represent the observed data points, and the green line is the calculated pattern. The cyan curve is the normalized error plot, and the red line is the background curve. The blue tick marks indicate the peak positions. The vertical scale has been multiplied by a factor of 20× for 2θ > 15.7°.

The final refinement of 136 variables using 28,101 observations and 91 restraints yielded the residuals *R*_wp_ = 0.0737 and GOF = 1.72. The largest peak (1.08 Å from C27) and hole (1.82 Å from N8) in the difference-Fourier map are 0.49 (11) and −0.46 (11) *e*Å^−3^, respectively. The final Rietveld plot is shown in Fig. 6 ▸. The largest features in the normalized error plot are in the positions and shapes of some of the strong low-angle peaks, and may indicate a change of the specimen during the measurement.

The crystal structure of avutometinib was optimized (fixed experimental unit cell) with density functional theory techniques using *VASP* (Kresse & Furthmüller, 1996 ▸) through the *MedeA* graphical inter­face (Materials Design, 2024 ▸). The calculation was carried out on 32 cores of a 144-core (768 Gb memory) HPE Superdome Flex 280 Linux server at North Central College. The calculation used the GGA-PBE functional, a plane wave cutoff energy of 400.0 eV, and a *k*-point spacing of 0.5 Å^−1^ leading to a 2 × 2 × 1 mesh, and took ∼1.1 h. Single-point density functional theory calculations (fixed experimental cell) and population analysis were carried out using *CRYSTAL23* (Erba *et al.*, 2023 ▸). (fixed experimental cell) and population analysis were carried out using *CRYSTAL17* (Dovesi *et al.*, 2018 ▸). The basis sets for the H, C, N and O atoms in the calculation were those of Gatti *et al.* (1994 ▸), and those for F and S were those of Peintinger *et al.* (2013 ▸). The calculations were run on a 3.5 GHz PC using 8 *k*-points and the B3LYP functional, and took ∼2.7 h. The powder pattern has been submitted to ICDD for inclusion in the Powder Diffraction File.

## Supplementary Material

Crystal structure: contains datablock(s) avutometinib, avutometinib_VASP. DOI: 10.1107/S2056989026004482/ee2030sup1.cif

Supporting information file. DOI: 10.1107/S2056989026004482/ee2030avutometinibsup2.cml

CCDC references: 2551067, 2551068

Additional supporting information:  crystallographic information; 3D view; checkCIF report

## Figures and Tables

**Table 1:** Hydrogen-bond geometry (Å, °)

*D*—H⋯*A*	*D*—H	H⋯*A*	*D*⋯*A*	*D*—H⋯*A*	Mulliken overlap	H-bond energy
N8—H44⋯O4^i^	1.04	1.95	2.961	165	0.054	5.4
N10—H45⋯O7^ii^	1.03	2.22	3.223	163	0.026	3.7
N10—H45⋯N9	1.03	2.49	2.977	108	0.011	–
C24—H41⋯O6^iii^	1.09	2.42	3.183	126	0.011	–
C33—H51⋯O6^iv^	1.09	2.35	3.170	131	0.015	–
C21—H39⋯N11^i^	1.09	2.80	3.882	170	0.012	–
C31—H49⋯N10^iv^	1.09	2.82	3.777	145	0.011	–

Symmetry codes: (i) *x* − 1, *y*, *z*; (ii) −*x*, −*y*, 1 − *z*; (iii) *x* + 1, *y*, *z*; (iv) *x* + 2, *y*, *z* − 1.

**Table 2:** Experimental details

	avutometinib
Crystal data	
Chemical formula	C_21_H_18_FN_5_O_5_S
*M*_r_	471.46
Crystal system, space group	Triclinic, *P* 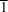
Temperature (K)	295
*a*, *b*, *c* (Å)	8.91097 (5), 9.47933 (5), 13.24641 (9)
α, β, γ (°)	83.9821 (4), 81.7759 (3), 66.67667 (9)
*V* (Å^3^)	1015.49 (1)
*Z*	2
Radiation type	Synchrotron, λ = 0.46873 Å
μ (mm^−1^)	0.02
Specimen shape, size (mm)	Cylinder, 2.0 × 1.5

Data collection	
Diffractometer	11-BM, APS
Specimen mounting	Kapton capillary
Data collection mode	Transmission
Scan method	Step
2θ values (°)	2θ_min_ = 0.510, 2θ_max_ = 49.995, 2θ_step_ = 0.001

Refinement	
*R* factors and goodness of fit	*R*_p_ = 0.058, *R*_wp_ = 0.072, *R*_exp_ = 0.043, *R*(*F*^2^) = 0.06083, χ^2^ = 2.941
No. of parameters	136
No. of restraints	91
(Δ/σ)_max_	1.795

Computer programs: *GSAS-II* (Toby & Von Dreele, 2013 ▸).

## supplementary crystallographic information

### 3-{[3-Fluoro-2-(methylsulfamoylamino)pyridin-4-yl]methyl}-4-methyl-7-(pyrimidin-2-yloxy)chromen-2-one (avutometinib) Crystal data

**Table:**

C_21_H_18_FN_5_O_5_S	γ = 66.67667 (9)°
*M**_r_* = 471.46	*V* = 1015.49 (1) Å^3^
Triclinic, *P*1	*Z* = 2
*a* = 8.91097 (5) Å	*D*_x_ = 1.542 Mg m^−^^3^
*b* = 9.47933 (5) Å	Synchrotron radiation, λ = 0.46873 Å
*c* = 13.24641 (9) Å	µ = 0.02 mm^−^^1^
α = 83.9821 (4)°	*T* = 295 K
β = 81.7759 (3)°	cylinder, 2.0 × 1.5 mm

### 3-{[3-Fluoro-2-(methylsulfamoylamino)pyridin-4-yl]methyl}-4-methyl-7-(pyrimidin-2-yloxy)chromen-2-one (avutometinib) Data collection

**Table:**

11-BM, APS diffractometer	Scan method: step
Specimen mounting: Kapton capillary	2θ_min_ = 0.510°, 2θ_max_ = 49.995°, 2θ_step_ = 0.001°
Data collection mode: transmission	

### 3-{[3-Fluoro-2-(methylsulfamoylamino)pyridin-4-yl]methyl}-4-methyl-7-(pyrimidin-2-yloxy)chromen-2-one (avutometinib) Refinement

**Table:**

Least-squares matrix: full	136 parameters
*R*_p_ = 0.058	91 restraints
*R*_wp_ = 0.072	27 constraints
*R*_exp_ = 0.043	Weighting scheme based on measured s.u.'s
*R*(*F*^2^) = 0.06083	(Δ/σ)_max_ = 1.795
49486 data points	Background function: Background function: "chebyschev-1" function with 6 terms: 39.70(6), -8.12(8), -5.86(7), 0.45(8), -3.44(7), -0.18(6), Background peak parameters: pos, int, sig, gam: 5.871(8), 5.48(8)e3, 4.80(12)e3, 0.100,
Profile function: Finger-Cox-Jephcoat function parameters U, V, W, X, Y, SH/L: peak variance(Gauss) = Utan(Th)^2^+Vtan(Th)+W: peak HW(Lorentz) = X/cos(Th)+Ytan(Th); SH/L = S/L+H/L U, V, W in (centideg)^2^, X & Y in centideg 1.163, -0.126, 0.063, 0.000, 0.000, 0.002,	Preferred orientation correction: Simple spherical harmonic correction Order = 2 Coefficients: 0:0:C(2,-2) = 0.0570; 0:0:C(2,-1) = -0.0160; 0:0:C(2,0) = -0.0620; 0:0:C(2,1) = -0.0340; 0:0:C(2,2) = 0.0470

### 3-{[3-Fluoro-2-(methylsulfamoylamino)pyridin-4-yl]methyl}-4-methyl-7-(pyrimidin-2-yloxy)chromen-2-one (avutometinib) Fractional atomic coordinates and isotropic or equivalent isotropic displacement parameters (Å^2^)

**Table:**

	*x*	*y*	*z*	*U*_iso_*/*U*_eq_	
S1	−0.24645 (18)	0.18626 (18)	0.42794 (12)	0.0421 (6)*	
F2	−0.0709 (3)	0.4497 (3)	0.18047 (17)	0.0346 (5)*	
O3	0.7085 (3)	0.3231 (4)	0.0934 (2)	0.0365 (4)*	
O4	0.5286 (3)	0.4613 (4)	0.2123 (2)	0.0365 (4)*	
O5	1.1225 (4)	0.0538 (4)	−0.1592 (2)	0.0466 (7)*	
O6	−0.4215 (3)	0.2499 (3)	0.4256 (2)	0.0429 (9)*	
O7	−0.1487 (3)	0.0326 (3)	0.4038 (2)	0.0429 (9)*	
N8	−0.1842 (3)	0.2993 (4)	0.3460 (2)	0.0346 (5)*	
N9	0.0824 (4)	0.1928 (4)	0.3953 (2)	0.0346 (5)*	
N10	−0.2097 (4)	0.2032 (3)	0.5409 (2)	0.0407 (13)*	
N11	1.3412 (4)	0.0840 (4)	−0.2574 (3)	0.0466 (7)*	
N12	1.0651 (3)	0.2462 (5)	−0.2842 (3)	0.0466 (7)*	
C13	0.4224 (3)	0.3609 (5)	0.0948 (3)	0.0365 (4)*	
C14	0.4605 (3)	0.2760 (5)	0.0115 (3)	0.0365 (4)*	
C15	0.2494 (3)	0.4418 (3)	0.1438 (3)	0.0346 (5)*	
C16	0.6307 (3)	0.2123 (5)	−0.0350 (3)	0.0365 (4)*	
C17	0.1945 (3)	0.3467 (5)	0.2286 (3)	0.0346 (5)*	
C18	0.7480 (3)	0.2434 (6)	0.0065 (3)	0.0365 (4)*	
C19	0.5500 (3)	0.3855 (5)	0.1392 (3)	0.0365 (4)*	
C20	0.3333 (4)	0.2424 (5)	−0.0335 (3)	0.0365 (4)*	
C21	0.6807 (4)	0.1290 (6)	−0.1232 (3)	0.0365 (4)*	
C22	0.9097 (4)	0.1913 (5)	−0.0337 (3)	0.0365 (4)*	
C23	0.0353 (3)	0.3584 (5)	0.2461 (3)	0.0346 (5)*	
C24	0.3001 (3)	0.2514 (5)	0.2970 (3)	0.0346 (5)*	
C25	0.9547 (4)	0.1156 (5)	−0.1231 (3)	0.0365 (4)*	
C26	0.8430 (4)	0.0789 (6)	−0.1659 (3)	0.0365 (4)*	
C27	−0.0221 (3)	0.2850 (5)	0.3307 (3)	0.0346 (5)*	
C28	0.2402 (4)	0.1795 (5)	0.3777 (3)	0.0346 (5)*	
C29	1.1788 (4)	0.1324 (5)	−0.2371 (3)	0.0466 (7)*	
C30	−0.2810 (6)	0.3515 (4)	0.5876 (3)	0.0407 (13)*	
C31	1.3927 (3)	0.1638 (6)	−0.3335 (4)	0.0466 (7)*	
C32	1.1255 (5)	0.3191 (5)	−0.3622 (3)	0.0466 (7)*	
C33	1.2907 (6)	0.2808 (6)	−0.3893 (3)	0.0466 (7)*	
H34	0.23891	0.55099	0.17829	0.0415*	
H35	0.16192	0.47507	0.08245	0.0415*	
H36	0.20524	0.32410	−0.00294	0.0438*	
H37	0.34758	0.11858	−0.01145	0.0438*	
H38	0.35062	0.25904	−0.12025	0.0438*	
H39	0.58774	0.10215	−0.16017	0.0438*	
H40	1.00374	0.21103	0.00682	0.0438*	
H41	0.43511	0.23367	0.28555	0.0437*	
H42	0.88436	0.00750	−0.23630	0.0438*	
H43	0.33257	0.10460	0.43208	0.0437*	
H44	−0.26680	0.38460	0.30380	0.0437*	
H45	−0.24990	0.13320	0.59250	0.0489*	
H46	−0.17896	0.37943	0.61243	0.0489*	
H47	−0.37075	0.34632	0.65705	0.0489*	
H48	−0.34902	0.44468	0.52986	0.0489*	
H49	1.52990	0.13446	−0.35462	0.0559*	
H50	1.03796	0.41730	−0.40887	0.0559*	
H51	1.34347	0.34168	−0.45476	0.0559*	

### 3-{[3-Fluoro-2-(methylsulfamoylamino)pyridin-4-yl]methyl}-4-methyl-7-(pyrimidin-2-yloxy)chromen-2-one (avutometinib) Geometric parameters (Å, º)

**Table:**

S1—O6	1.4366 (19)	C22—C25	1.376 (2)
S1—O7	1.413 (2)	C22—H40	1.140 (3)
S1—N8	1.640 (2)	C23—F2	1.353 (2)
S1—N10	1.6130 (18)	C23—C17	1.365 (2)
F2—C23	1.353 (2)	C23—C27	1.3987 (18)
O3—C18	1.369 (2)	C24—C17	1.386 (2)
O3—C19	1.3724 (18)	C24—C28	1.365 (3)
O4—C19	1.213 (3)	C24—H41	1.136 (2)
O5—C25	1.403 (3)	C25—O5	1.403 (3)
O5—C29	1.363 (3)	C25—C22	1.376 (2)
O6—S1	1.4366 (19)	C25—C26	1.383 (3)
O7—S1	1.413 (2)	C26—C21	1.384 (3)
N8—S1	1.640 (2)	C26—C25	1.383 (3)
N8—C27	1.383 (2)	C26—H42	1.140 (3)
N8—H44	1.030 (3)	C27—N8	1.383 (2)
N9—C27	1.338 (2)	C27—N9	1.338 (2)
N9—C28	1.349 (3)	C27—C23	1.3987 (18)
N10—S1	1.6130 (18)	C28—N9	1.349 (3)
N10—C30	1.459 (2)	C28—C24	1.365 (3)
N10—H45	1.029 (3)	C28—H43	1.140 (3)
N11—C29	1.3283 (17)	C29—O5	1.363 (3)
N11—C31	1.335 (3)	C29—N11	1.3283 (17)
N12—C29	1.3248 (17)	C29—N12	1.3248 (17)
N12—C32	1.349 (3)	C30—N10	1.459 (2)
C13—C14	1.357 (2)	C30—H46	1.140 (4)
C13—C15	1.506 (2)	C30—H47	1.140 (4)
C13—C19	1.457 (3)	C30—H48	1.140 (5)
C14—C13	1.357 (2)	C31—N11	1.335 (3)
C14—C16	1.459 (2)	C31—C33	1.351 (3)
C14—C20	1.504 (2)	C31—H49	1.140 (3)
C15—C13	1.506 (2)	C32—N12	1.349 (3)
C15—C17	1.515 (2)	C32—C33	1.371 (3)
C15—H34	1.140 (3)	C32—H50	1.140 (3)
C15—H35	1.140 (3)	C33—C31	1.351 (3)
C16—C14	1.459 (2)	C33—C32	1.371 (3)
C16—C18	1.388 (2)	C33—H51	1.140 (3)
C16—C21	1.397 (2)	H34—C15	1.140 (3)
C17—C15	1.515 (2)	H35—C15	1.140 (3)
C17—C23	1.365 (2)	H36—C20	1.140 (4)
C17—C24	1.386 (2)	H37—C20	1.140 (5)
C18—O3	1.369 (2)	H38—C20	1.140 (4)
C18—C16	1.388 (2)	H39—C21	1.140 (3)
C18—C22	1.369 (3)	H40—C22	1.140 (3)
C19—O3	1.3724 (18)	H41—C24	1.136 (2)
C19—O4	1.213 (3)	H42—C26	1.140 (3)
C19—C13	1.457 (3)	H43—C28	1.140 (3)
C20—C14	1.504 (2)	H44—N8	1.030 (3)
C20—H36	1.140 (4)	H45—N10	1.029 (3)
C20—H37	1.140 (5)	H46—C30	1.140 (4)
C20—H38	1.140 (4)	H47—C30	1.140 (4)
C21—C16	1.397 (2)	H48—C30	1.140 (5)
C21—C26	1.384 (3)	H49—C31	1.140 (3)
C21—H39	1.140 (3)	H50—C32	1.140 (3)
C22—C18	1.369 (3)	H51—C33	1.140 (3)
			
O6—S1—O7	121.73 (17)	H36—C20—H38	109.5 (4)
O6—S1—N8	103.46 (18)	H37—C20—H38	109.5 (3)
O7—S1—N8	107.88 (19)	C16—C21—C26	120.21 (16)
O6—S1—N10	108.15 (18)	C16—C21—H39	120.0 (2)
O7—S1—N10	105.89 (17)	C26—C21—H39	119.8 (3)
N8—S1—N10	109.38 (17)	C18—C22—C25	118.60 (17)
C18—O3—C19	122.01 (12)	C18—C22—H40	120.0 (2)
C25—O5—C29	118.2 (3)	C25—C22—H40	121.4 (2)
S1—N8—C27	122.28 (18)	F2—C23—C17	117.97 (14)
S1—N8—H44	120.1 (2)	F2—C23—C27	119.41 (14)
C27—N8—H44	117.7 (2)	C17—C23—C27	122.57 (11)
C27—N9—C28	117.74 (16)	C17—C24—C28	119.33 (12)
S1—N10—C30	121.0 (2)	C17—C24—H41	120.0 (2)
S1—N10—H45	109.5 (2)	C28—C24—H41	120.7 (3)
C30—N10—H45	103.5 (3)	O5—C25—C22	117.7 (3)
C29—N11—C31	114.62 (10)	O5—C25—C26	120.7 (3)
C29—N12—C32	114.34 (10)	C22—C25—C26	120.73 (16)
C14—C13—C15	123.60 (17)	C21—C26—C25	119.86 (16)
C14—C13—C19	120.69 (10)	C21—C26—H42	120.1 (3)
C15—C13—C19	115.64 (17)	C25—C26—H42	120.1 (3)
C13—C14—C16	119.32 (10)	N8—C27—N9	119.01 (15)
C13—C14—C20	122.09 (13)	N8—C27—C23	121.04 (14)
C16—C14—C20	118.57 (13)	N9—C27—C23	119.86 (10)
C13—C15—C17	114.1 (2)	N9—C28—C24	124.01 (15)
C13—C15—H34	109.5 (2)	N9—C28—H43	120.0 (3)
C17—C15—H34	107.1 (3)	C24—C28—H43	116.0 (3)
C13—C15—H35	108.7 (3)	O5—C29—N11	116.0 (3)
C17—C15—H35	108.7 (2)	O5—C29—N12	116.1 (3)
H34—C15—H35	108.7 (2)	N11—C29—N12	127.94 (13)
C14—C16—C18	118.57 (10)	N10—C30—H46	109.4 (4)
C14—C16—C21	123.50 (15)	N10—C30—H47	109.5 (4)
C18—C16—C21	117.81 (14)	H46—C30—H47	109.5 (3)
C15—C17—C23	121.66 (17)	N10—C30—H48	109.4 (3)
C15—C17—C24	121.79 (17)	H46—C30—H48	109.5 (4)
C23—C17—C24	116.40 (10)	H47—C30—H48	109.5 (4)
O3—C18—C16	121.12 (11)	N11—C31—C33	123.81 (16)
O3—C18—C22	116.32 (15)	N11—C31—H49	120.0 (4)
C16—C18—C22	122.50 (15)	C33—C31—H49	116.2 (4)
O3—C19—O4	115.99 (17)	N12—C32—C33	122.95 (16)
O3—C19—C13	118.12 (12)	N12—C32—H50	120.0 (4)
O4—C19—C13	125.85 (17)	C33—C32—H50	117.1 (4)
C14—C20—H36	109.5 (2)	C31—C33—C32	116.29 (17)
C14—C20—H37	109.5 (4)	C31—C33—H51	120.0 (4)
H36—C20—H37	109.5 (3)	C32—C33—H51	123.7 (4)
C14—C20—H38	109.5 (3)		

### (avutometinib_VASP) Crystal data

**Table:**

C_21_H_18_FN_5_O_5_S	α = 83.98°
*M**_r_* = 471.46	β = 81.78°
Triclinic, *P*1	γ = 66.68°
*a* = 8.91097 Å	*V* = 1015.53 Å^3^
*b* = 9.47933 Å	*Z* = 2
*c* = 13.24641 Å	

### (avutometinib_VASP) Data collection

**Table:**

*h* = →	*l* = →
*k* = →	

### (avutometinib_VASP) Fractional atomic coordinates and isotropic or equivalent isotropic displacement parameters (Å^2^)

**Table:**

	*x*	*y*	*z*	*B*_iso_*/*B*_eq_	
S1	−0.24757	0.18753	0.42895		
F2	−0.07477	0.45568	0.17671		
O3	0.71646	0.31732	0.09192		
O4	0.53929	0.45121	0.21599		
O5	1.12701	0.05321	−0.16336		
O6	−0.42292	0.24813	0.42629		
O7	−0.14823	0.03113	0.40340		
N8	−0.19006	0.30945	0.34680		
N9	0.07524	0.19945	0.39447		
N10	−0.21091	0.20419	0.54292		
N11	1.34192	0.08760	−0.25877		
N12	1.06472	0.25390	−0.28664		
C13	0.42895	0.35914	0.09446		
C14	0.46617	0.27131	0.01093		
C15	0.25568	0.44005	0.14351		
C16	0.63371	0.20853	−0.03693		
C17	0.19668	0.34780	0.22776		
C18	0.75384	0.23729	0.00578		
C19	0.55739	0.38083	0.13895		
C20	0.33632	0.24377	−0.03426		
C21	0.68522	0.12449	−0.12629		
C22	0.91658	0.18843	−0.03742		
C23	0.03348	0.36349	0.24311		
C24	0.30019	0.24992	0.29808		
C25	0.96096	0.10896	−0.12596		
C26	0.84706	0.07495	−0.17096		
C27	−0.02552	0.28926	0.32727		
C28	0.23404	0.17983	0.37918		
C29	1.17844	0.13683	−0.24060		
C30	−0.28532	0.35499	0.58704		
C31	1.39657	0.16416	−0.33617		
C32	1.12400	0.32658	−0.36442		
C33	1.29130	0.28551	−0.39322		
H34	0.24832	0.54394	0.17773		
H35	0.16996	0.48068	0.08520		
H36	0.22340	0.27002	0.01837		
H37	0.37818	0.12413	−0.05544		
H38	0.30606	0.31650	−0.10496		
H39	0.59597	0.09911	−0.16153		
H40	1.00632	0.21209	−0.00181		
H41	0.42946	0.23167	0.29139		
H42	0.88621	0.01237	−0.24077		
H43	0.31035	0.10618	0.43672		
H44	−0.27345	0.36968	0.29517		
H45	−0.08805	0.14370	0.55085		
H46	−0.22639	0.43396	0.55260		
H47	−0.27350	0.33849	0.66903		
H48	−0.41643	0.40770	0.57712		
H49	1.52997	0.12683	−0.35303		
H50	1.03244	0.42144	−0.40429		
H51	1.33943	0.34281	−0.45717		

### (avutometinib_VASP) Hydrogen-bond geometry (Å, º)

**Table:**

*D*—H···*A*	*D*—H	H···*A*	*D*···*A*	*D*—H···*A*
N8—H44···O4^i^	1.036	1.947	2.961	165.2
N10—H45···O7^ii^	1.030	2.222	3.223	163.4
N10—H45···N9	1.030	2.491	2.977	108.1
C24—H41···O6^iii^	1.087	2.417	3.183	126.3
C33—H51···O6^iv^	1.087	2.350	3.170	130.9
C21—H39···N11^i^	1.091	2.803	3.882	170.3
C31—H49···N10^iv^	1.094	2.826	3.777	145.3

Symmetry codes: (i) *x*−1, *y*, *z*; (ii) −*x*, −*y*, −*z*+1; (iii) *x*+1, *y*, *z*; (iv) *x*+2, *y*, *z*−1.

## References

[bb1] Antao, S. M., Hassan, I., Wang, J., Lee, P. L. & Toby, B. H. (2008). *Can. Mineral.* **46**, 1501–1509.

[bb2] Bernstein, J., Davis, R. E., Shimoni, L. & Chang, N. L. (1995). *Angew. Chem. Int. Ed. Engl.* **34**, 1555–1573.

[bb3] K. Brandenburg, K. & Putz, H. (2025). *DIAMOND V 5.1.1*. Crystal Impact, Bonn, Germany.

[bb4] Bravais, A. (1866). *Etudes Cristallographiques.* Paris: Gauthier Villars.

[bb5] Bruno, I. J., Cole, J. C., Kessler, M., Luo, J., Motherwell, W. D. S., Purkis, L. H., Smith, B. R., Taylor, R., Cooper, R. I., Harris, S. E. & Orpen, A. G. (2004). *J. Chem. Inf. Comput. Sci.* **44**, 2133–2144. 10.1021/ci049780b 15554684

[bb6] Dassault Systèmes. (2024). *BIOVIA Materials Studio 2025*. BIOVIA, San Diego, CA

[bb7] Donnay, J. D. H. & Harker, D. (1937). *Am. Mineral.* **22**, 446-467.

[bb37] Dovesi, R., Erba, A., Orlando, R., Zicovich-Wilson, C. M., Civalleri, B., Maschio, L., Rérat, M., Casassa, S., Baima, J., Salustro, S. & Kirtman, B. (2018). *WIREs Comput. Mol. Sci.* **8**, e1360.

[bb8] Erba, A., Desmarais, J. K., Casassa, S., Civalleri, B., Donà, L., Bush, I. J., Searle, B., Maschio, L., Edith-Daga, L., Cossard, A., Ribaldone, C., Ascrizzi, E., Marana, N. L., Flament, J.-P. & Kirtman, B. (2023). *J. Chem. Theory Comput.* **19**, 6891–6932. 10.1021/acs.jctc.2c00958 PMC10601489 36502394

[bb9] Etter, M. C. (1990). *Acc. Chem. Res.* **23**, 120–126.

[bb10] Favre-Nicolin, V. & Černý, R. (2002). *J. Appl. Cryst.* **35**, 734–743.

[bb11] Friedel, G. (1907). *Bull. Soc. Française Minéral.* **30**, 326–455.

[bb12] Gatti, C., Saunders, V. R. & Roetti, C. (1994). *J. Chem. Phys.* **101**, 10686–10696.

[bb13] Groom, C. R., Bruno, I. J., Lightfoot, M. P. & Ward, S. C. (2016). *Acta Cryst.* B**72**, 171–179. 10.1107/S2052520616003954 PMC4822653 27048719

[bb14] Hirshfeld, F. L. (1977). *Theor. Chim. Acta* **44**, 129–138.

[bb15] Kabekkodu, S., Dosen, A. & Blanton, T. N. (2024). *Powder Diffr.* **39**, 47–59.

[bb16] Kaduk, J. A., Crowder, C. E., Zhong, K., Fawcett, T. G. & Suchomel, M. R. (2014). *Powder Diffr.* **29**, 269–273.

[bb17] Kaduk, J. A., Dosen, A. & Blanton, T. N. (2025). *Powder Diffr.* **40**, 168–174.

[bb18] Kim, S., Chen, J., Cheng, T., Gindulyte, A., He, J., He, S., Li, Q., Shoemaker, B. A., Thiessen, P. A., Yu, B., Zaslavsky, L., Zhang, J. & Bolton, E. E. (2023). *Nucleic Acids Res.* **51**, D1373–D1380. 10.1093/nar/gkac956 PMC9825602 36305812

[bb19] Kresse, G. & Furthmüller, J. (1996). *Comput. Mater. Sci.* **6**, 15–50.

[bb20] Lee, P. L., Shu, D., Ramanathan, M., Preissner, C., Wang, J., Beno, M. A., Von Dreele, R. B., Ribaud, L., Kurtz, C., Antao, S. M., Jiao, X. & Toby, B. H. (2008). *J. Synchrotron Rad.* **15**, 427–432. 10.1107/S0909049508018438 18728312

[bb21] Macrae, C. F., Sovago, I., Cottrell, S. J., Galek, P. T. A., McCabe, P., Pidcock, E., Platings, M., Shields, G. P., Stevens, J. S., Towler, M. & Wood, P. A. (2020). *J. Appl. Cryst.* **53**, 226–235. 10.1107/S1600576719014092 PMC6998782 32047413

[bb22] Materials Design. (2024). *MedeA 3.7.2*. Materials Design Inc., San Diego, USA.

[bb23] MDI. (2025). *JADE Pro version 9.3*. Materials Data, Livermore, USA.

[bb24] Motherwell, W. D. S., Shields, G. P. & Allen, F. H. (2000). *Acta Cryst.* B**56**, 857–871. 10.1107/S0108768100007230 11006562

[bb25] O’Boyle, N. M., Banck, M., James, C. A., Morley, C., Vandermeersch, T. & Hutchison, G. R. (2011). *J. Cheminform* **3**, 33. 10.1186/1758-2946-3-33 PMC3198950 21982300

[bb26] Peintinger, M. F., Oliveira, D. V. & Bredow, T. (2013). *J. Comput. Chem.* **34**, 451–459. 10.1002/jcc.23153 23115105

[bb27] Spackman, P. R., Turner, M. J., McKinnon, J. J., Wolff, S. K., Grimwood, D. J., Jayatilaka, D. & Spackman, M. A. (2021). *J. Appl. Cryst.* **54**, 1006–1011. 10.1107/S1600576721002910 PMC8202033 34188619

[bb28] Stephens, P. W. (1999). *J. Appl. Cryst.* **32**, 281–289.

[bb31] Streek, J. van de & Neumann, M. A. (2014). *Acta Cryst.* B**70**, 1020–1032. 10.1107/S2052520614022902 PMC4468513 25449625

[bb29] Sykes, R. A., McCabe, P., Allen, F. H., Battle, G. M., Bruno, I. J. & Wood, P. A. (2011). *J. Appl. Cryst.* **44**, 882–886. 10.1107/S0021889811014622 PMC3246811 22477784

[bb30] Toby, B. H. & Von Dreele, R. B. (2013). *J. Appl. Cryst.* **46**, 544–549.

[bb32] Vibha, K., Prachality, N. C., Reddy, R. A., Ravikantha, M. N. & Thipperudrappa, J. (2023). *Chem. Phys. Impact* **6**, 100147.

[bb33] Wang, J., Toby, B. H., Lee, P. L., Ribaud, L., Antao, S. M., Kurtz, C., Ramanathan, M., Von Dreele, R. B. & Beno, M. A. (2008). *Rev. Sci. Instrum.* **79**, 085105. 10.1063/1.2969260 19044378

[bb34] Wavefunction (2025). *Spartan ’24. V. 1.3.1.* Wavefunction Inc., Irvine, USA.

[bb35] Wheatley, A. M. & Kaduk, J. A. (2019). *Powder Diffr.* **34**, 35–43.

[bb36] Whitfield, P. S. (2025). 18th Pharmaceutical Powder X-ray Diffraction Symposium, Cambridge UK.

